# The Tasmanian devil microbiome—implications for conservation and management

**DOI:** 10.1186/s40168-015-0143-0

**Published:** 2015-12-21

**Authors:** Yuanyuan Cheng, Samantha Fox, David Pemberton, Carolyn Hogg, Anthony T. Papenfuss, Katherine Belov

**Affiliations:** Faculty of Veterinary Science, RMC Gunn Building, University of Sydney, Sydney, New South Wales 2006 Australia; Department of Primary Industries, Parks, Water and Environment, 134 Macquarie Street, Hobart, Tasmania 7000 Australia; Zoo and Aquarium Association, Mosman, New South Wales 2088 Australia; Bioinformatics Division, The Walter and Eliza Hall Institute of Medical Research, Parkville, Victoria 3052 Australia; Department of Medical Biology, University of Melbourne, Melbourne, Victoria 3010 Australia

**Keywords:** Tasmanian devil, Marsupial, Carnivore, Microbiota, Endangered species, Conservation and management

## Abstract

**Background:**

The Tasmanian devil, the world’s largest carnivorous marsupial, is at risk of extinction due to devil facial tumour disease (DFTD), a fatal contagious cancer. The Save the Tasmanian Devil Program has established an insurance population, which currently holds over 600 devils in captive facilities across Australia. Microbes are known to play a crucial role in the health and well-being of humans and other animals, and increasing evidence suggests that changes in the microbiota can influence various aspects of host physiology and development. To improve our understanding of devils and facilitate management and conservation of the species, we characterised the microbiome of wild devils and investigated differences in the composition of microbial community between captive and wild individuals.

**Results:**

A total of 1,223,550 bacterial 16S ribosomal RNA (rRNA) sequences were generated via Roche 454 sequencing from 56 samples, including 17 gut, 15 skin, 18 pouch and 6 oral samples. The devil’s gut microbiome was dominated by Firmicutes and showed a high Firmicutes-to-Bacteroidetes ratio, which appears to be a common feature of many carnivorous mammals. Metabolisms of carbohydrates, amino acids, energy, cofactors and vitamins, nucleotides and lipids were predicted as the most prominent metabolic pathways that the devil's gut flora contributed to. The microbiota inside the female’s pouch outside lactation was highly similar to that of the skin, both co-dominated by Firmicutes and Proteobacteria. The oral microbiome had similar proportions of Proteobacteria, Bacteroidetes, Firmicutes and Fusobacteria.

**Conclusions:**

Compositional differences were observed in all four types of microbiota between devils from captive and wild populations. Certain captive devils had significantly lower levels of gut bacterial diversity than wild individuals, and the two groups differed in the proportion of gut bacteria accounting for the metabolism of glycan, amino acids and cofactors and vitamins. Further studies are underway to investigate whether alterations in the microbiome of captive devils can have impacts on their ability to adapt and survive following re-introduction to the wild.

**Electronic supplementary material:**

The online version of this article (doi:10.1186/s40168-015-0143-0) contains supplementary material, which is available to authorized users.

## Background

The Tasmanian devil (*Sarcophilus harrisii*; “devil” hereinafter) is the largest remaining carnivorous marsupial and is now restricted to the island state of Tasmania, Australia. The species is at risk of extinction due to a fatal contagious cancer called devil facial tumour disease (DFTD), which was first reported in 1996 and has since reduced the devil population size by about 86 % [1, 2]. In light of this significant population decline, urgent conservation management approaches have been undertaken. The Save the Tasmanian Devil Program has established an insurance population, currently consisting of over 600 devils kept in a range of intensive management facilities and free-range enclosures throughout Australia [3]. The aim of this program is to capture and retain the genetic diversity of the species until the risk of extinction is gone. This population will be used to repopulate the wild if local extinctions occur and to supplement wild populations at risk of inbreeding due to population crashes.

A large amount of effort has gone into characterising the genome of the Tasmanian devil [4, 5], yet the “second genome” of the devil—its microbiome—has remained uncharacterised. The microbiome is known to play a crucial role in human health and welfare [6]. The composition of microbial community and alterations in its structure have been associated with diabetes [7], inflammatory bowel disease [8], rheumatoid arthritis [9], asthma [10], obesity [11], susceptibility to infections [12] and response to cancer immunotherapies [13]. Besides the extensive research conducted on humans [11, 14], the importance of the microbiome is also known for livestock [15, 16] and companion animals [17], and recently, some work has also been carried out on wildlife species [18–22]. Here we focus on the microbiome of the Tasmanian devil.

Devils have a natural longevity of 5 to 6 years. Similar in size to a small dog, an adult devil (>2 years) on average measures around 60 cm long with a 25-cm tail and 30 cm high at the shoulder [23]. The weight ranges between 7.7–13 kg in males and 4.5–9 kg in females [24]. Devils are generally nocturnal; they search for food between sunset and sunrise and spend most of the day in a den [25]. Being dominantly a scavenger, devils feed largely on carrion of animals, such as possums, wallabies, kangaroos and wombats, though they have also evolved to be able to consume and digest a wide variety of food, such as fish, insect, fruit and vegetation [25]. Devils also predate and have been recorded killing possums, pademelons, wombats, birds and invertebrates including spiders and large gum moths. Communal feeding and the use of communal latrines are commonly observed in devils, which is unusual for an animal perceived to be solitary [25, 26]. Like other marsupials, devils have very short gestation, which usually lasts only 18 days [25]. Twenty or more underdeveloped imps are born, but no more than four (two to three on average) can survive, as a female devil has only four teats in her pouch [25]. Devils are found across Tasmania and occupy a large variety of habitats, ranging from coastal scrub to rainforests to alpine areas [27]. However, due to ease of feeding and burrowing, grazing land, open forest, open woodland and coastal scrub are preferred habitat types for devils rather than dense wet eucalypt, heath, open grassland and bleak rocky areas [23, 28].

In this study, we characterised the composition of bacterial communities at four body sites of devils, including gut (faecal), mouth, skin surface and inside the pouch. Also, we investigated whether there are differences in the microbiome between devils in the wild and in captivity.

## Methods

### Sample collection and ethics

Three oral, 12 pouch and 9 skin swab and 11 faecal samples were collected from 23 wild devils in 4 different areas in Tasmania, including Granville Harbour, Bronte, Takone and Narawntapu National Park (metadata in Additional file 1; map in Additional file 2). This was conducted during routine monitoring trips by the Save the Tasmanian Devil Program, coordinated by the Tasmanian Department of Primary Industries, Parks, Water and Environment. Devils were trapped, health-checked and swabbed following standard veterinary protocols. Faecal samples were collected from the base of the trap or hessian sack if voided during the capture and sampling process. Another three oral, six pouch, six skin and six faecal samples were collected from eight insurance population animals kept in captivity in New South Wales. Captive devils were handled, and samples were collected by the keepers. Sample collection procedures were approved by the Animal Ethics Committee of the University of Sydney (permit #2013/6039 and #2014/550: sampling from captive devils; #681: sampling from wild devils).

### Microbial DNA extraction, sequencing and analysis

Swab and faecal samples were stored at −80 °C prior to processing. Microbial genomic DNA was extracted from faecal samples using QIAamp DNA Stool Mini Kit (Qiagen) and from swabs using QIAamp UCP Pathogen Mini Kit (Qiagen). Barcoded amplicons of the 16S ribosomal RNA (rRNA) gene V1-V3 region (27F-519R) were generated and sequenced on a Roche 454 GS FLX System by the Australian Genome Research Facility Ltd (Brisbane). Sequence data was processed and analysed using the QIIME (v1.9) pipelines [29]. Raw reads were demultiplexed and quality-filtered using default parameters. To determine the most suitable method for operational taxonomic unit (OTU) picking, three commonly used strategies, de novo, closed-reference and open-reference, were tested on a subset of data comprising 108,047 sequences from oral samples. At the 97 % similarity cut-off level, 82.7 % of sequences failed to match the latest release of Greengenes (13_8) 99 % OTU reference dataset (with reverse strand matching enabled). This led to a substantial underestimate of OTU numbers using the closed-reference method, while similar numbers of OTUs were produced with the open-reference (where sequences that did not hit a reference at a certain identity threshold level were subsequently clustered de novo) and de novo methods (Additional file 3). In order to retain sequences that do not match the reference database with high similarity and maintain consistency with other microbiome studies in wildlife species [18–22, 30], the de novo method was employed with OTUs defined as sequences with >97 % similarity. OTUs were then aligned to the Greengenes (13_8) 97 % OTU database and assigned taxonomy (using QIIME default value 0.9 for minimum similarity).

Baseline characterisation of the composition of bacterial communities in devils was performed using data from the wild individuals. Within-sample phylotype richness (alpha diversity) and dissimilarity between samples (beta diversity) were calculated on rarefied OTU tables for the following comparisons: (1) between all samples and (2) for each microbiota type, between different geographic locations. The minimum number of sequences per sample was used for rarefaction, that is, 8585 for gut samples, 15,242 for mouth, 9685 for pouch, 6745 for skin and 6745 for overall sample comparison (Additional file 1). In addition to principal coordinates analysis (PCoA), unsupervised clustering using the unweighted pair group method with arithmetic mean (UPGMA) analysis of distance between samples was conducted on the total sample set to produce a dendrogram with bootstrap support values. Monte Carlo method (999 permutations) was used to evaluate significance of differences in alpha diversity and UniFrac distances (both weighted and unweighted) between populations. Wilcoxon rank sum tests were performed to identify OTUs that showed significantly different frequencies between wild and captive devils.

Metabolic profiles of the gut microbiome (11 wild and 7 captive samples) as determined by KEGG pathways were predicted using the package PICRUSt 1.0 [31]. OTUs were re-picked against the Greengenes 13_5 database, which is utilised by PICRUSt, at 90 % identity, and sequences that failed to hit the reference were excluded from subsequent functional prediction. The average Nearest Sequenced Taxon Index (NSTI) indicating accuracy of PICRUSt predictions was 0.09 ± 0.01, which is within the common range estimated for mammalian guts [31]. Wilcoxon rank sum tests were used to determine significant differences in the metabolic profile between wild and captive samples.

## Results and discussion

### Dataset general description

The dataset described in this study is available in the MG-RAST database under project number 14948. Fifty-six microbiota samples, including 17 gut (11 wild and 6 captive), 15 skin (9 wild, 6 captive), 18 pouch (12 wild, 6 captive) and 6 oral (3 wild, 3 captive) samples, were sequenced at 16S rRNA gene V1-V3 region on a Roche 454 GS FLX System. The dataset contains a total of 1,223,550 sequences with the average length of 488 bp. The number of sequences per sample ranges between 6745 and 71,862. Metadata of the samples, including the numbers of reads and operational taxonomic units (OTUs) and information on the animals, are provided in Additional file 1.

### Taxonomic composition of Tasmanian devil microbiomes

**Fig. 1:**
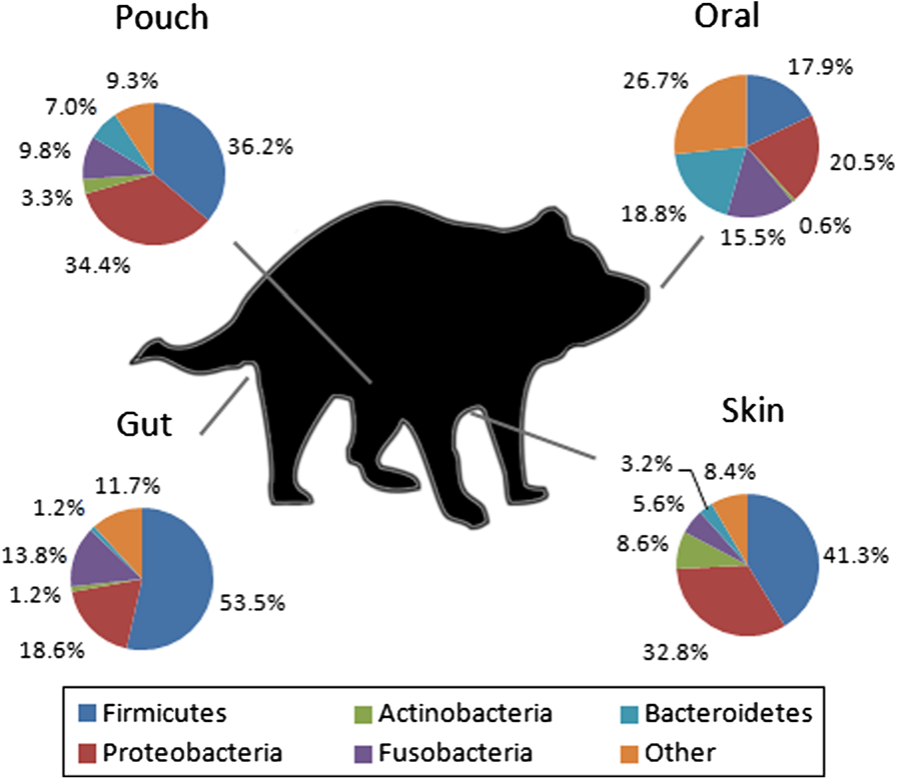
Baseline characterisation of gut, skin, pouch and oral microbiome in the Tasmanian devil. The image of the devil was adapted from the logo of the Save the Tasmanian Devil Program (http://www.tassiedevil.com.au/)

**Fig. 2:**
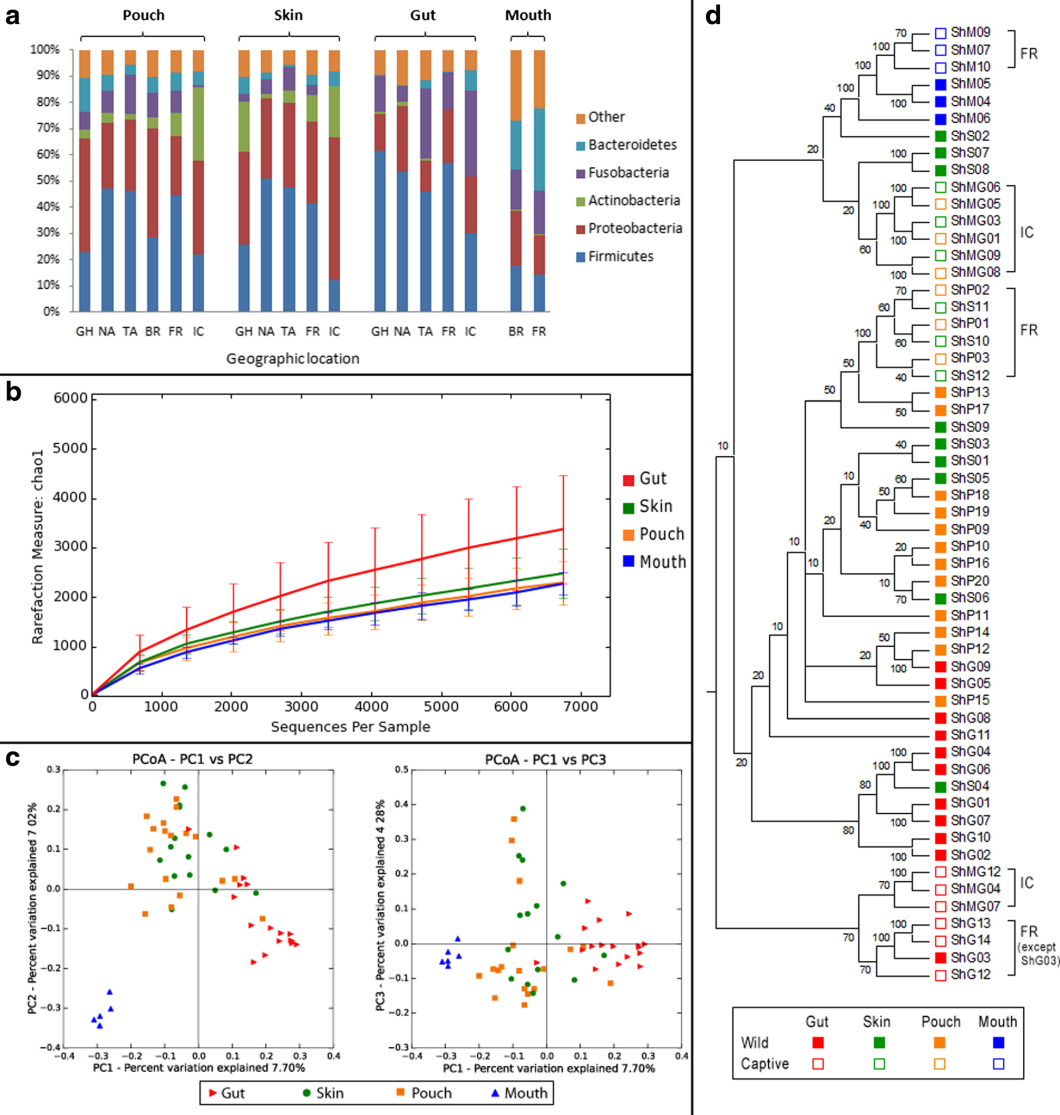
Overall comparisons between gut, skin, pouch and oral microbiome. **a** Composition of bacterial community at phylum level. **b** Phylotype richness inferred using Chao1 metric with *error bars* showing the standard deviation of each sample set. **c** PCoA of unweighted UniFrac distances across all samples. **d** UPGMA tree with bootstrap support values inferring confidence for each node

Members of 39 bacterial phyla were detected across all samples, with Firmicutes, Proteobacteria, Fusobacteria, Bacteroidetes and Actinobacteria revealed as the top five most prevalent phyla in the microbiota present in Tasmanian devils. The average compositions of bacterial communities at the four examined body sites in wild devils are summarised in Fig. 1 (see also Fig. 2a for compositions at different geographic locations; relative abundance of taxonomic groups in each sample provided in Additional file 4).

**Fig. 3:**
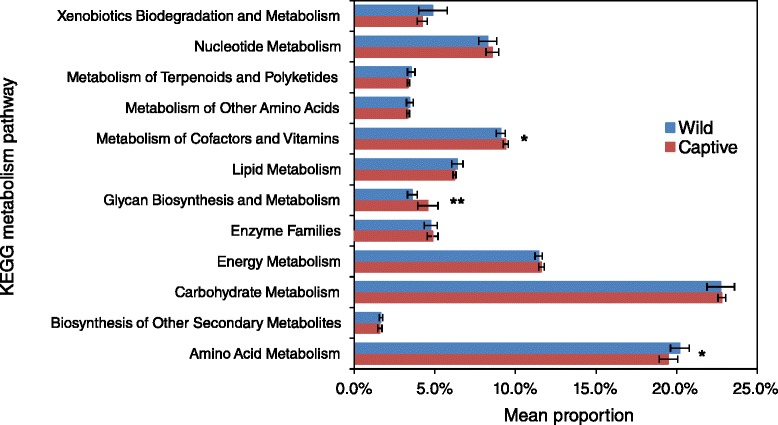
Predicted metabolic functions of the gut flora of wild and captive devils (asterisks indicate significant difference between the two groups: **, p < 0.05; *, p < 0.1)

The devil’s faecal microbiome had significantly higher phylotype richness than the other three studied microbiome types (Fig. 2b), with an average of 2817 OTUs identified in each sample. The faecal microbiota of wild devils (sample size *N* = 11) was dominated by Firmicutes, which showed a high relative abundance of 53.5 ± 3.9 % (Fig. 1). Under this phylum, Clostridium (18.5 ± 2.4 % of total sequences) was identified as the most common bacteria in devil faecal samples. This genus is known to contain species that are normal components of human intestinal flora with protein decomposition activities, as well as some important pathogens that can release toxins and cause intestinal diseases [32–35]. A large variety of Proteobacteria were detected in devil faeces, making up a significant proportion (18.6 ± 3.5 %; 71.7 % of which were Gammaproteobacteria and 22.1 % Alphaproteobacteria) of the gut microbiome. This level of Proteobacteria is higher than that found in the gut of many other mammalian species (Table 1; on average 8.8 % in mammals according to [35]). Fusobacteria, mostly belonging to genera Cetobacterium (9.2 ± 4.8 %) and Fusobacterium (4.6 ± 1.8 %), comprised 13.8 ± 4.5 % of the devil faecal flora. Compared to many other mammals that have been investigated, one distinctive characteristic of the devil gut microbiome is the low prevalence of Bacteroidetes (1.2 ± 0.6 %), which have been found to account for 5.5–19.8 % of koala (*Phascolarctos cinereus*), 15.0 % of red kangaroo (*Macropus rufus*), 36.1 % of cat (*Felis catus*), 31–34 % of dog (*Canis familiaris*) and 16.9 % of human faecal bacteria (Table 1). Interestingly, such low abundance of Bacteroidetes has also been observed in the gut microbiota of a few other carnivorous mammals, such as the cheetah (*Acinonyx jubatus*), spotted hyena (*Crocuta crocuta*) and polar bear (*Ursus maritimus*) [35]. Despite differences in the relative abundance of some other bacterial phyla between these animals, a low level of Bacteroidetes appears to be a common feature, which may be related to their carnivorous or scavenger diet preference. Also, it has been found in humans and mice that high abundance of Firmicutes and low Bacteroidetes (the “obese microbiome”) is associated with high efficiency in energy harvest from the diet and a greater chance of the individual to develop obesity [11]. In the wild, devils can gorge up to 40 % of their body mass in a single meal and then not feed for 2 to 3 days [36]. Therefore, the observed high ratio of gut Firmicutes to Bacteroidetes in the devil could also possibly be attributed to the need to efficiently extract and store energy from occasionally limited food sources. Functional predictions of the devil gut flora as determined by KEGG pathways [37] are shown in Fig. 3. Among the 12 primary metabolism pathways, carbohydrate (22.7 %), amino acid (20.2 %), energy (11.4 %), cofactor and vitamin (9.1 %), nucleotide (8.3 %) and lipid (6.4 %) metabolisms were inferred as the most prominent categories in the predicted metabolic profile.

**Table 1:** Comparison of gut, oral and skin flora composition between species (only common taxa with >1 % abundance are shown)

Microbiota/species			Bacterial phylum									
			Firmicutes	Bacteroidetes	Actinobacteria	Proteobacteria	Fusobacteria	Synergistetes	Verrucomicrobia	Spirochaetes	SR1	Cyanobacteria
Gut (faecal)	Human [61]		79.4 %	16.9 %	2.50 %	1 %						
	Cat [62]		36.3 %	36.1 %	7.7 %	12.4 %						
	Dog [63]		14–28 %	31–34 %	0.8–1.4 %	5–7 %	23–40 %					
	Koala [22]		62.9–86.9 %	5.5–19.8 %		2.2–6.0 %	0.00–3.6 %	0.45–6.1 %				
	Red kangaroo [35]		68.6 %	15.0 %		7.0 %			9.4 %			
	Tasmanian devil		53.5 %	1.2 %	1.2 %	18.6 %	13.8 %					
Oral	Human [64]		36.7 %	17.3 %	11.6 %	17.1 %	5.2 %			7.9 %		
	Cat [65]		6.7 %	9.3 %		75.2 %	1.3 %			1.8 %	2.7 %	
	Dog [66]		45.9 %	12.2 %	3.4 %	14.7 %	2.8 %	3.7 %		10.5 %		
	Koala [21]			26.1–40.6 %		30.4–50.9 %	0.00–5.9 %					
	Tasmanian devil		17.9 %	18.8 %		20.5 %	15.5 %					
Skin	Human [38]	Dry sites	12 %	14 %	28 %	41 %						
		Moist sites	25 %	9 %	36 %	26 %						
	Dog [39]	Dorsal lumbar	13.8 %	1.4 %	11.5 %	61.2 %						1.5 %
	Tasmanian devil	Abdomen	41.3 %	3.2 %	8.6 %	32.8 %	5.6 %					
		Pouch	36.2 %	7.0 %	3.3 %	34.4 %	9.8 %

The mammalian skin microbiota varies across different sites of the body [38, 39]. In this study, we examined the dry site in the chest-abdomen area of nine wild devils, revealing a microbiota co-dominated by Firmicutes (41.3 ± 4.6 %) and Proteobacteria (32.8 ± 3.1 %) (Fig. 1). Of the detected Firmicutes, 50.3 % were Clostridia and 47.2 % Bacilli, while 93.8 % of the Proteobacteria belonged to the Gamma subdivision (Additional file 4). Other relatively abundant (>1 % of all skin sequences) bacterial groups found on the devil’s skin included Actinobacteria (8.6 ± 3.1 %), Fusobacteria (5.6 ± 2.3 %) and Bacteroidetes (3.2 ± 1.6 %).

The female’s pouch, which is essentially a fold of skin on the abdomen that covers the teats, is a unique and important feature of many marsupials. During lactation, it provides a protective environment for the underdeveloped neonates which are born without an adaptive immune system [40]. Our results suggested that the microbiota inside the devil’s pouch was highly similar to that of the skin. Firstly, the pouch and skin samples showed similar levels of phylotype richness (Fig. 2b), with comparable numbers of OTUs identified (on average, 1907 per sample in the pouch and 1828 in the skin). Secondly, they shared similar overall taxonomic compositions (Fig. 1), with the pouch flora also co-dominated by Firmicutes (36.2 ± 3.6 %) and Proteobacteria (34.4 ± 4.5 %), though there were more Clostridia (77.1 % of detected Firmicutes) and fewer Bacilli (21.6 %) in the pouch, and levels of Fusobacteria (9.8 ± 2.0 %) and Bacteroidetes (7.0 ± 1.8 %) were slightly higher and Actinobacteria (3.3 ± 0.7 %) lower than those of the skin. Thirdly, pouch and skin were grouped together in the principal coordinates analysis (PCoA) of unweighted UniFrac distances across all samples, separate from the majority of gut and oral samples (Fig. 2c). Unsupervised clustering of samples revealed that matching pouch and skin samples collected from the same individual clustered together, demonstrating clearly a close relationship between the pouch and skin microbiota (see “FR” and “IC” samples in Fig. 2d). However, this may only be the case in non-lactating devils. Previous research in koalas has shown that during lactation, the mother secretes peptides with antimicrobial activities in the pouch, which may serve as a mechanism to protect the joeys from harmful microbes [41]. Similar immune protection of pouch young through antimicrobial peptides has also been observed in the tammar wallaby (*Macropus eugenii*) [42]. How antimicrobial peptides in the pouch secretions and the milk may impact the composition of pouch flora is an important and interesting area for further investigation.

The microbial composition of the devil’s oral flora is also of particular interest because zoo keepers have reported that devil bites cause severe infections that are difficult to treat. We detected an average of 1505 OTUs per oral sample, comprising similar proportions of Proteobacteria (20.5 ± 3.4 %), Bacteroidetes (18.8 ± 4.4 %), Firmicutes (17.9 ± 2.4 %) and Fusobacteria (15.5 ± 3.2 %) (Fig. 1). Spirochaetes have been found to be relatively common in the oral cavity of humans (7.9 %), cats (1.8 %) and dogs (10.5 %) (Table 1), but turned out to be rare in devils (0.013 ± 0.007 %; Additional file 4). A large number of unclassified sequences (using default taxonomy assignment settings) were detected in devil mouth samples, accounting for 25.9 ± 5.8 % of the total oral microbiota, which may indicate that the devil’s mouth harbours uncommon microbes that may represent novel taxa [43]. By lowering the minimum similarity threshold to assign taxonomy to a sequence using the UCLUST method [44] to 0.85, an estimation of the composition of these unclassified taxa (intotal, 12,468 sequences) was produced (Additional file 5), suggesting that 50.0 % sequences were most similar to Proteobacteria (Pseudomonadales) and 30.7 % Fusobacteria (Fusobacteriales).

### Comparison between captive and wild devils: implications for conservation

We compared samples collected from different geographic locations to investigate whether the microbiota varies between sites, especially between devil populations in the wild and in captivity (Additional file 2). Wild sampling sites included Granville Harbour (GH for abbreviation hereinafter), Bronte (BR), Takone (TA) and Narawntapu (NA). TA and BR are inland areas belonging to different bioregions (TA, Tasmanian Northern Slopes; BR, Tasmanian Southern Ranges) [45], whereas NA and GH are two coastal areas with distinct biogeographic features (GH described as cold dolerite wet and NA warm sandy dry). Captive samples were collected from an intensive captive (IC) and a free-range (FR) devil holding facility from the mainland of Australia. The recommended diet for captive devils includes whole or partial carcasses of rabbit, emu, beef, wallaby/kangaroo, venison, rats, mice, 1-day-old chicks/adult chicks, duck and fish, and the suggested feeding regime includes fast and gorge feeds to mimic their natural feeding patterns [46].

**Fig. 4:**
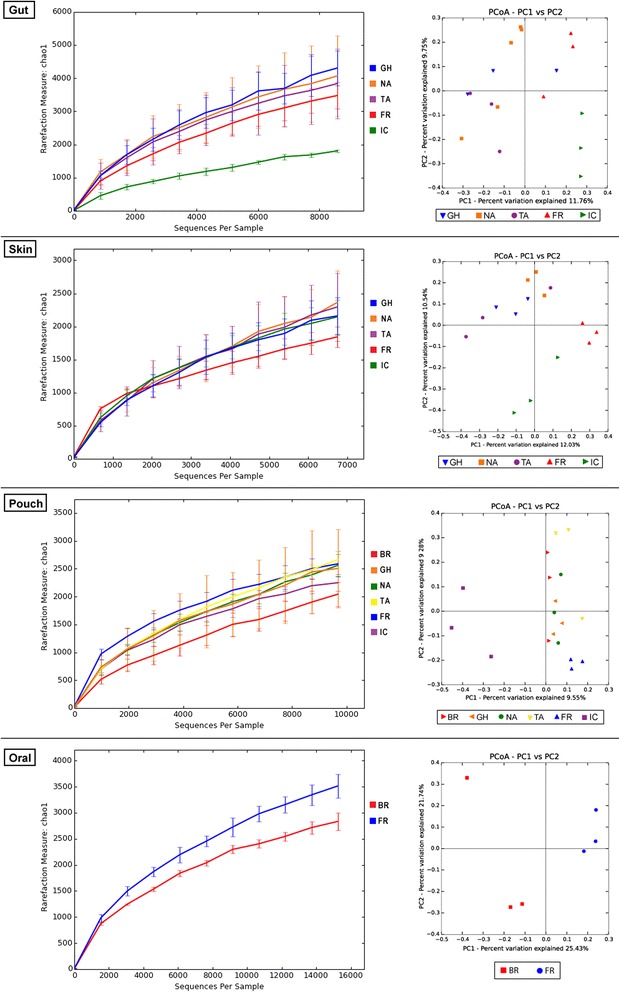
Comparison of devil microbiome between different geographic sites. In each panel, the *left* graph shows the phylotype richness, while the *right* graph shows the PCoA of unweighted UniFrac distances between sites

The gut microbiome was surveyed in three wild sites (NA, GH and TA) and both captive sites (Fig. 4). The wild devils showed similar levels of phylotype richness and no significant difference between the within- and between-group UniFrac distances (both unweighted and weighted), suggesting that the composition of gut microbiome of a wild devil was not affected by the geographic origin of the animal (Additional files 6 and 7). Compared to the wild devils, however, statistically significant differences were found in the examined captive population, particularly in the IC samples (Monte Carlo test *p* < 0.05). As shown in Fig. 4, IC devils not only showed high dissimilarity to all wild groups (unweighted distance 0.89 ± 0.00; detailed statistics of unweighted and weighted distances provided in Additional file 7) but also had a significantly lower level of gut bacterial richness than other examined devils. At the genus level, 70 OTUs were detected to show significantly different relative abundance between wild and captive populations (Wilcoxon rank sum test *p* < 0.05; Additional file 8), with the two most pronounced differences found in the level of Cetobacterium (9.21 % in wild devils vs. 0.02 % in captive devils) and Epulopiscium (wild 1.08 vs. captive 8.06 %). Cetobacterium and Epulopiscium species have been previously found in the intestinal tracts of other animals, such as humans [47] and fish [48, 49], though their functions are not yet fully understood. In the predicted metabolic profiles (Fig. 3), captive and wild devils showed a significant difference (*p* < 0.05) in the proportion of microbiome accounting for the glycan biosynthesis and metabolism pathway (wild 3.6 vs. captive 4.6 %); other less prominent differences (*p* < 0.1) included metabolisms of amino acids (20.2 vs. 19.5 %) and cofactors and vitamins (9.1 vs. 9.4 %).

Compared to the gut, the skin and pouch microbiota appeared to be more dependent on the geographic location, as a relatively higher degree of separation was seen between certain wild groups, such as between NA and GH skin samples (Fig. 4; Additional file 6). However, statistically significant distinctions were still mostly found between the captive and the wild. Among all sampling sites, FR devils had the highest inter-individual similarity in their skin and pouch flora, reflected as low within-group distances (skin unweighted 0.70 ± 0.01 and pouch 0.71 ± 0.01; Additional file 7), resulting in small clusters on the PCoA plots. Also can be seen on the PCoA plots is that the captive samples, especially the IC samples, were well separated from the wild ones, with the average unweighted distance being 0.86 ± 0.01 in the skin and 0.871 ± 0.003 in pouch. The relative abundance of 159 skin and 115 pouch OTUs was detected to have changed significantly in captive devils compared to those in the wild (Additional file 8). Forty-one of these changes occurred in both types of microbiota; for instance, Brochothrix comprised on average 7.38 % of skin bacterial community and 2.71 % of pouch in wild devils, but were rarely found (<0.01 %) in the examined captive devils. The level of Mycobacterium in the skin was significantly higher in the captive samples (Additional file 8), which may be related to the reported mycobacterial skin infections in devils from different captive facilities [46, 50].

Oral microbiome comparison was performed between one wild (BR) and one captive (FR) sampling site. As shown in Fig. 4, the oral bacterial community of FR devils had a higher level of species diversity than that of wild individuals. Also, the within-group UniFrac distance of FR samples were markedly lower than the distance between FR and BR groups (Additional files 6 and 7), again, suggesting significant compositional distinction between the oral flora of captive and wild devils. Among the 35 OTUs that showed statistically significant differences in frequency between the two populations (Additional file 8), the most drastic change was observed in Porphyromonas (wild 10.5 vs. captive 23.9 %), a genus known to contain pathogenic members such as *Porphyromonas gingivalis*, which is a major causative agent of chronic periodontitis in humans [51].

In summary, our results suggest that devils in captivity tend to have a different microbiome composition to individuals in the wild. This finding is not unexpected, and similar observations have been made previously in other species, such as the giant panda (*Ailuropoda melanoleuca*) [19], red panda (*Ailurus fulgens*) [18] and black howler monkey (*Alouatta pigra*) [20]. The microbiome has a dynamic nature and can be influenced by a large variety of factors, such as nutrition, medical treatment, environmental and social conditions and stress [52, 53]. Although zoo animals are often provided with food and environmental enrichment that imitate their natural diet and habitat, it is usually inevitable that the artificial settings in captivity can still cause behavioural or physiological changes in animals. It has been found that the microbiome of herbivorous animals can be relatively stable and remains similar between captive and wild individuals [21, 54]. However, our results demonstrated that at least in carnivorous species, microbiome alterations can represent a major physiological change of animals in captivity. We also observed a trend that between the two captive groups, FR devils were relatively less different than the wild group than IC devils (Fig. 4), suggesting free-range enclosure to be a more preferable option for microbiome management in devils.

It is uncertain whether the detected changes in the captive devil’s microbiome can have an adverse impact on the health of the animals. One thing that may have an unfavourable effect and needs to be noted is the low diversity of gut microbiome observed in some captive individuals, which can result in increased risks of obesity [55] and thus in turn leads to reduced success rate of captive breeding. Also, the microbiome is known to play a crucial role in shaping the host’s immune and endocrine systems [56, 57]. Studies conducted on germ-free mouse models revealed that the lab mice are more susceptible to certain bacterial, viral and parasitic infections than wild mice due to immunological defects (reviewed in [58]). Therefore, another important question to investigate in future follow-up studies is whether the wild-type microbiome will be restored in devils after they are returned to the wild. Since 2012, the Save the Tasmanian Devil Program has started to release insurance devils onto a disease-free island, as a trial run for future re-introductions of devils back into the wild [59, 60]. Monitoring how the microbiome changes when captive devils are released into the wild will provide further insights on the dynamics of microbiome and its role in the adaptive success and long-term survival of devils following re-introduction.

## Conclusions

Here, we report the first comprehensive microbiota characterisation using next-generation sequencing in a carnivorous marsupial. Our results revealed that the bacterial communities present in the gut, mouth, skin and pouch of Tasmanian devils are primarily comprised of Firmicutes, Proteobacteria, Fusobacteria, Bacteroidetes and Actinobacteria. Devils have a highly diverse gut microbiome, which has a high Firmicutes-to-Bacteroidetes ratio. The gut flora contributed to a range of metabolism pathways, with carbohydrate, amino acid, energy, cofactor and vitamin and nucleotide and lipid metabolisms predicted as the most prominent categories. The microbiota inside the female’s pouch outside lactation is highly similar to that on the skin, both co-dominated by Firmicutes and Proteobacteria. Differences were found between the microbiome of devils from captive and wild populations. Further investigations, including temporal monitoring of the same animals before and after releasing to the wild, are underway to study whether alterations in the microbiome of captive devils can have long-term impacts on their survival in the wild.

## Availability of data and materials

Data of 56 microbiota samples described in this study is available in the MG-RAST database under project number 14948 and NCBI SRA database with accession number SRP063696.

## Additional files

Additional file 1:
**Metadata of all 56 microbiome samples.**
Additional file 2:
**Sampling sites.** Maps were adapted from Google Maps. Additional file 3:
**Comparison of OTU picking methods (using a subset of data comprising 108,047 sequences).**
Additional file 4:
**Taxonomic composition of 56 microbiome samples at phylum, class, order, family and genus levels.**
Additional file 5:
**Estimated taxonomic composition (0.85 similarity cut-off level) of oral sequences with low similarity to the reference dataset.**
Additional file 6:
**Additional figures of microbiota comparisons between different geographic locations.**
Additional file 7:
**Unweighted and weighted UniFrac distances between samples from different geographic locations.**
Additional file 8:
**List of OTUs that showed significantly different frequencies between wild and captive devils. Taxa with relative abundance over 1 % in either wild or captive group are highlighted.**

## Abbreviations

BR: Bronte
FR: free range
GH: Granville Harbour
IC: intensive captive
NA: Narawntapu National Park
OTU: operational taxonomic unit
TA: Takone
