# Supplementary figures and images for: The Tasmanian devil microbiome—implications for conservation and management
Source: Microbiome. 2015 Dec 21;3:76. doi: 10.1186/s40168-015-0143-0 (PMC4687321; doi:10.1186/s40168-015-0143-0)

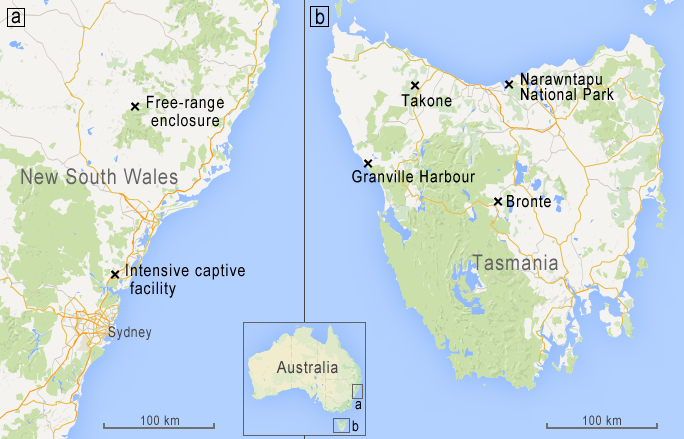

Supplement: Additional file 2: — Sampling sites. Maps were adapted from Google Maps. [file 40168_2015_143_MOESM2_ESM.tif]

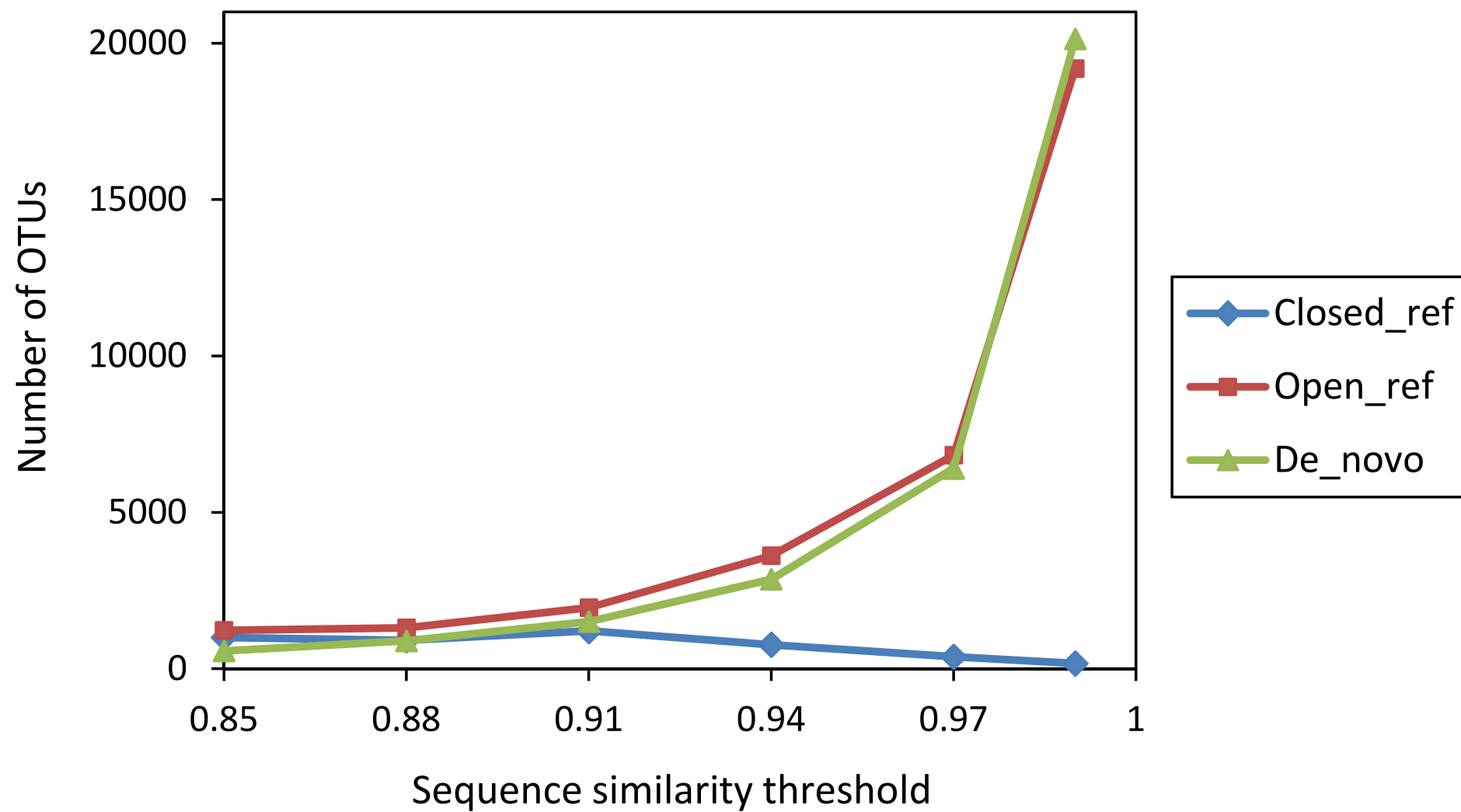

Supplement: Additional file 3: — Comparison of OTU picking methods (using a subset of data comprising 108,047 sequences). [file 40168_2015_143_MOESM3_ESM.pdf]
