# Supplementary material for: The Tasmanian devil microbiome—implications for conservation and management
Source: Microbiome. 2015 Dec 21;3:76. doi: 10.1186/s40168-015-0143-0 (PMC4687321; doi:10.1186/s40168-015-0143-0)
Supplement: Additional file 5: — Estimated taxonomic composition (0.85 similarity cut-off level) of oral sequences with low similarity to the reference dataset. [file 40168_2015_143_MOESM5_ESM.pdf]

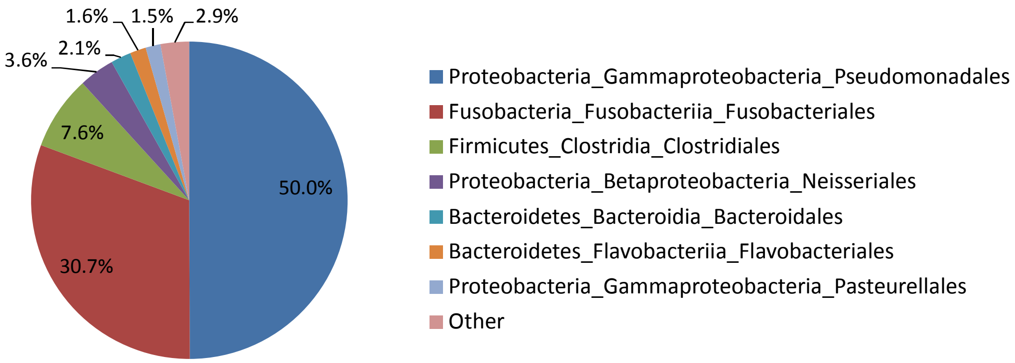
1.6%
1.5%
2.9%
2.1%
3.6%
7.6%
50.0%
30.7%
Proteobacteria_Gammaproteobacteria_Pseudomonadales
Fusobacteria_Fusobacteriia_Fusobacteriales
Firmicutes_Clostridia_Clostridiales
Proteobacteria_Betaproteobacteria_Neisseriales
Bacteroidetes_Bacteroidia_Bacteroidales
Bacteroidetes_Flavobacteriia_Flavobacteriales
Proteobacteria_Gammaproteobacteria_Pasteurellales
Other
