# Supplementary material for: The Tasmanian devil microbiome—implications for conservation and management
Source: Microbiome. 2015 Dec 21;3:76. doi: 10.1186/s40168-015-0143-0 (PMC4687321; doi:10.1186/s40168-015-0143-0)
Supplement: Additional file 6: — Additional figures of microbiota comparisons between different geographic locations. [file 40168_2015_143_MOESM6_ESM.pdf]

Additional file. Comparison of Tasmanian devil microbiota between different geographic sites
(a) Pair-wise unweighted UniFrac distances between sample groups.
(b) PCoA analysis of unweighted UniFrac distances.

Page 2 - gut
Page 3 - skin
Page 4 - pouch
Page 5 - oral

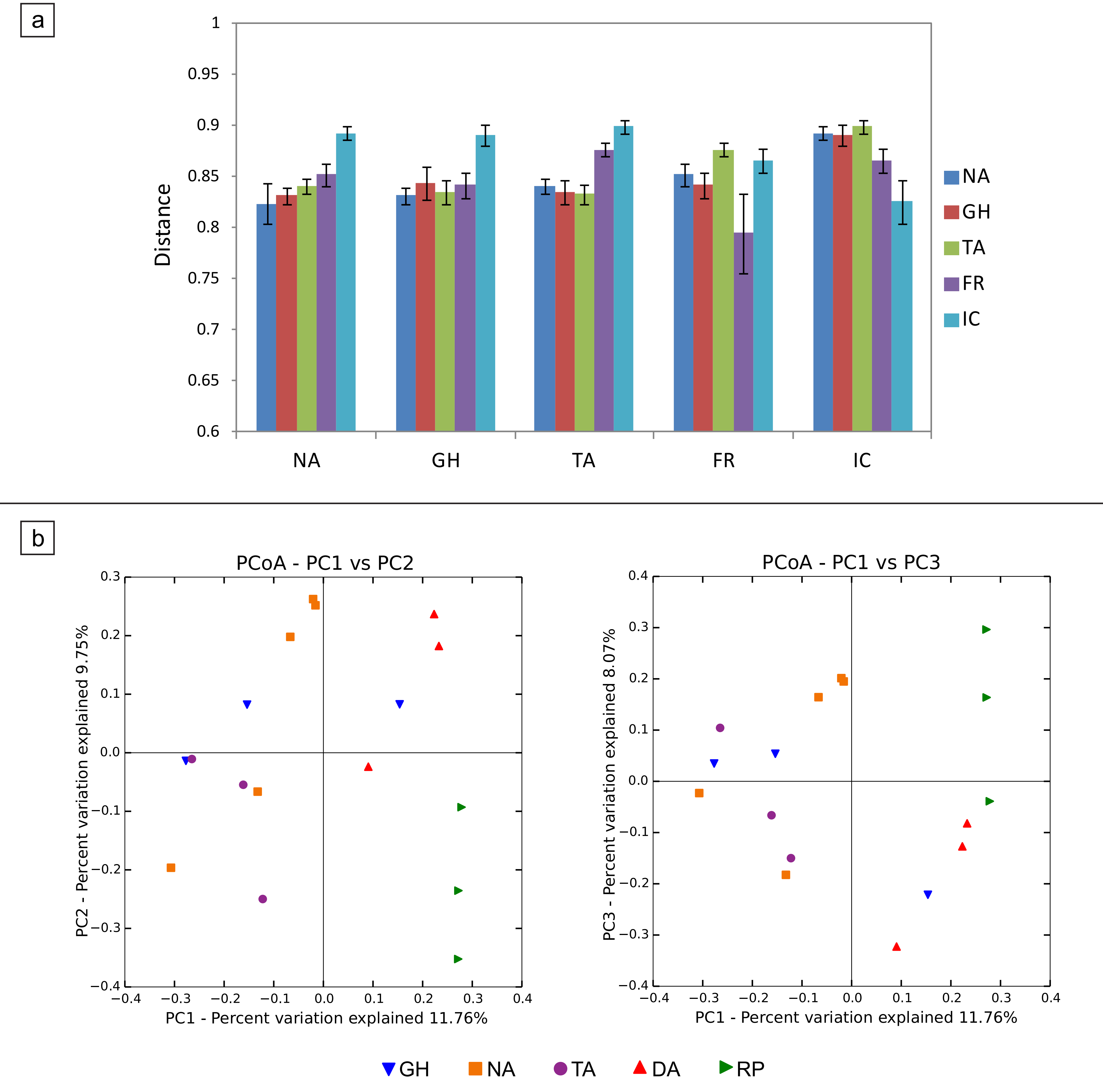
a
Distance
1
0.95
0.9
0.85
0.8
0.75
0.7
0.65
0.6
NA
GH
TA
FR
IC
NA
GH
TA
FR
IC
b
PCoA - PC1 vs PC2
PC2 - Percent variation explained 9.75%
PC1 - Percent variation explained 11.76%
PCoA - PC1 vs PC3
PC3 - Percent variation explained 8.07%
PC1 - Percent variation explained 11.76%
GH
NA
TA
DA
RP

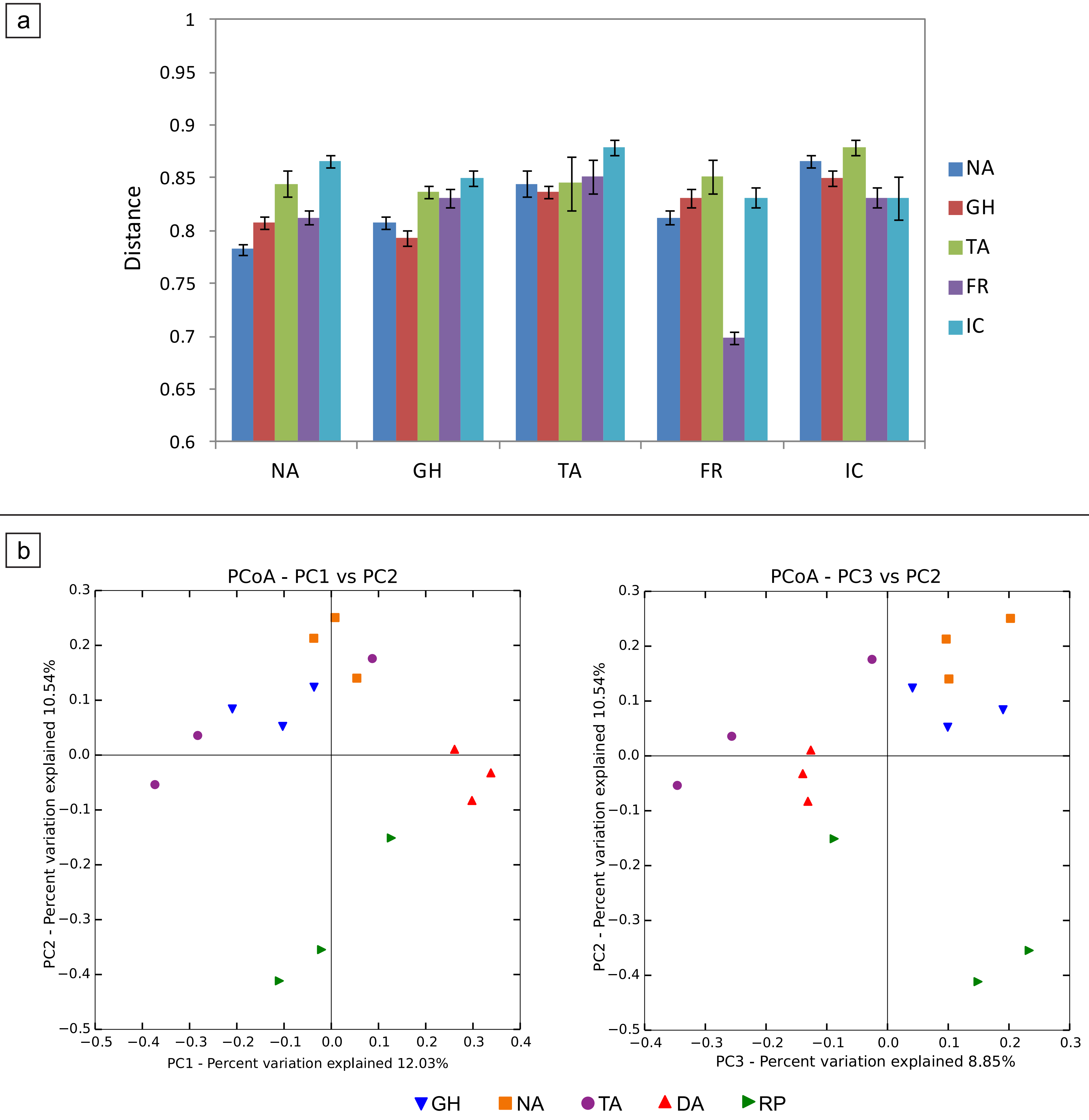
a
Distance
1
0.95
0.9
0.85
0.8
0.75
0.7
0.65
0.6
NA
GH
TA
FR
IC
NA
GH
TA
FR
IC
b
PCoA - PC1 vs PC2
PC2 - Percent variation explained 10.54%
PC1 - Percent variation explained 12.03%
PCoA - PC3 vs PC2
PC2 - Percent variation explained 10.54%
PC3 - Percent variation explained 8.85%
0.3
0.2
0.1
0.0
−0.1
−0.2
−0.3
−0.4
−0.5
−0.5
−0.4
−0.3
−0.2
−0.1
0.0
0.1
0.2
0.3
0.4
−0.4
−0.3
−0.2
−0.1
0.0
0.1
0.2
0.3
GH
NA
TA
DA
RP

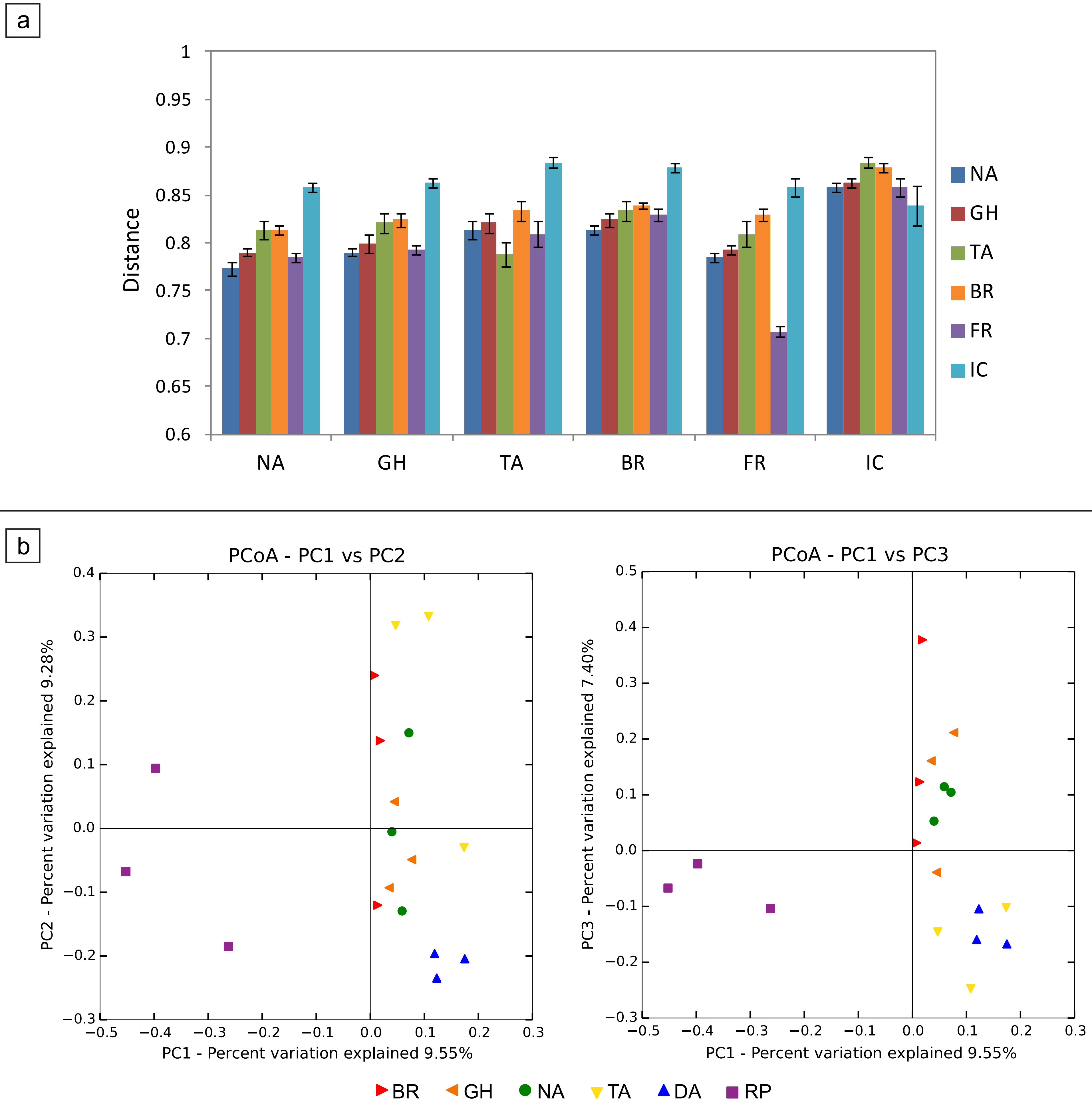
a
Distance
1
0.95
0.9
0.85
0.8
0.75
0.7
0.65
0.6
NA
GH
TA
BR
FR
IC
NA
GH
TA
BR
FR
IC
b
PCoA - PC1 vs PC2
PC2 - Percent variation explained 9.28%
PC1 - Percent variation explained 9.55%
PCoA - PC1 vs PC3
PC3 - Percent variation explained 7.40%
PC1 - Percent variation explained 9.55%
0.5
0.4
0.3
0.2
0.1
0.0
−0.1
−0.2
−0.3
−0.5
−0.4
−0.3
−0.2
−0.1
0.0
0.1
0.2
0.3
BR
GH
NA
TA
DA
RP

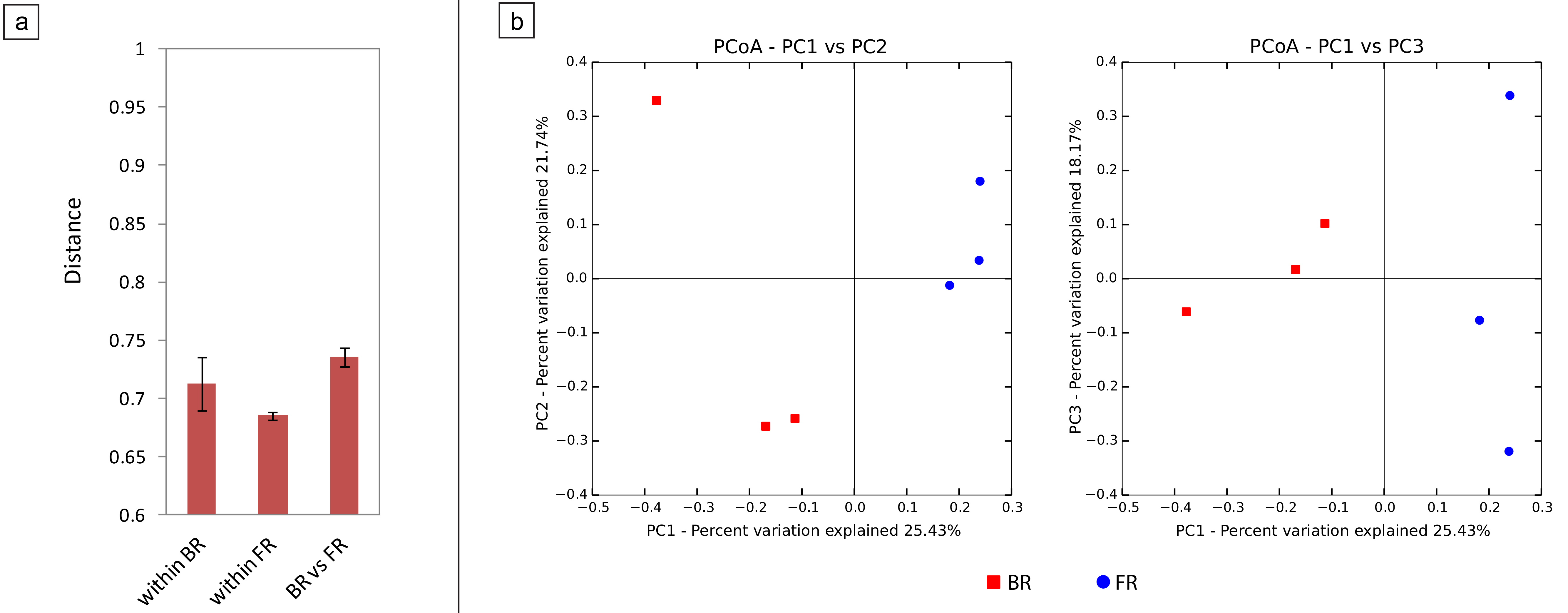

a
Distance
1
0.95
0.9
0.85
0.8
0.75
0.7
0.65
0.6
within BR
within FR
BR vs FR
b
PCoA - PC1 vs PC2
PC2 - Percent variation explained 21.74%
PC1 - Percent variation explained 25.43%
PCoA - PC1 vs PC3
PC3 - Percent variation explained 18.17%
PC1 - Percent variation explained 25.43%
0.4
0.3
0.2
0.1
0.0
−0.1
−0.2
−0.3
−0.4
−0.5
−0.4
−0.3
−0.2
−0.1
0.0
0.1
0.2
0.3
BR
FR
